# A cross-sectional analysis of patient-based records on mortality among patients with diabetic ketoacidosis in a tertiary hospital in Ghana

**DOI:** 10.4314/gmj.v59i2.1

**Published:** 2025-06

**Authors:** Yacoba Atiase, Josephine Akpalu, Ernest Yorke, Margaret Reynolds, Ofoliquaye Allotey Annan, Robert Aryee, Alfred Yawson

**Affiliations:** 1 Department of Medicine and Therapeutics, University of Ghana Medical School, Accra, Ghana; 2 Department of Medicine and Therapeutics, Korle Bu Teaching Hospital, Accra, Ghana; 3 Department of Physiology, University of Ghana Medical School, Accra, Ghana; 4 Department of Anaesthesia, University of Ghana Medical School, Accra, Ghana; 5 Department of Community Health, University of Ghana Medical School, Accra, Ghana; 6 Department of Cardiology, University of Ghana Medical Centre, Legon, Ghana; 7 National Diabetes Management and Research Centre, Accra, Ghana

**Keywords:** Diabetic ketoacidosis, Diabetic emergencies, Africa, Ghana, mortality, outcomes

## Abstract

**Objectives:**

To determine the mortality of patients admitted and managed for Diabetic ketoacidosis (DKA) at a teaching hospital

**Design:**

A cross-sectional study of the medical records of all 70 patients 18 years and older, managed for DKA in the adult emergency room of Korle-Bu Teaching Hospital in Ghana from March to July 2019.

**Setting:**

The study was conducted among adult patients managed for diabetic ketoacidosis in the adult emergency room and adult medical wards of Korle-Bu Teaching Hospital.

**Participants:**

The participants were patients aged 18 years or older who were admitted with diabetic ketoacidosis.

**Interventions:**

Patients managed for diabetic ketoacidosis in the adult emergency room of Korle-Bu Teaching Hospital in Ghana had their outcomes assessed.

**Main outcome measures:**

Outcomes of DKA management, including mortality, discharge, duration to either of these outcomes and associations were measured.

**Results:**

The mortality rate from DKA in this study was 15.7%. Mean age, duration of diabetes, and blood glucose at admission were 44.06(±16.23) years, 7.19(±6.04) years, and 26.37(±6.70) mmol/L, respectively. Female gender and pulse rate >100 or <60 beats/minute were independently associated with mortality. Only 2 out of 70 (2.85%) patients were managed in the Intensive Care Unit (ICU).

**Conclusion:**

The Mortality of DKA management in this hospital was high (15.7%). Most of these patients (97.15%) were not managed in the ICU or HDU; to improve this high mortality, there is a need to use the guidelines in the diagnosis, categorisation and management of DKA and to make the ICU and HDU available, accessible and affordable in our centre and elsewhere in Ghana.

**Funding:**

None declared

## Introduction

The commonest acute complication of diabetes is diabetic ketoacidosis (DKA), which is associated with high mortality even in developed countries.^1^ A diagnosis of DKA is made in the presence of 1) blood glucose > 11.0mmol/l;2)ketonemia >/3mmol/l or significant ketonuria more than 2+ on standard urine sticks; 3) and bicarbonate levels (HCO3) <15mmol/L and/or venous pH <7.3.^2^

The pathophysiology of DKA is complex and multifactorial, resulting from a relative or absolute insulin deficiency that leads to high blood glucose levels. This is due to a reduction in glucose uptake by tissues, such as muscles, and an increase in counterregulatory hormones, including glucagon, cortisol, growth hormone, and epinephrine.

Hyperglycemia causes the passage of large amounts of urine, resulting in dehydration and a profound loss of electrolytes, such as potassium, a characteristic of DKA. The absence of insulin, together with the increased counterregulatory hormones, promotes the breakdown of fat as an alternative source of energy.^3^ ^4^ ^5^, resulting in the formation and accumulation of ketone bodies, including acetone, acetoacetate, and beta-hydroxybutyrate (BHB), which cause ketonemia and acidemia. As the body's intracellular buffer system is exceeded, these ketone bodies spill over into urine for excretion, resulting in ketonuria. BHB, the predominant ketone body in circulation, is increased by 10 times during an episode of DK^6^, but it is not water-soluble and undetected in urine.

Generally, DKA should be managed in intensive care units (ICU) or at least in a high dependency unit (HDU), particularly when DKA is considered severe, as it is associated with higher mortality. DKA mortality is declining appreciably in many centres across the globe^7^, with current studies showing a mortality rate of up to 1.8%.^8^,^9^ However, in many low-income countries in sub-Saharan Africa, including Ghana, DKA mortality remains high.^10^ In Libya, the mortality of DKA measured over 12 months was 11.7%^11^, in Nigeria, 18%^12^, 29.8% in Kenya,^10^ and 0.6% in Libya.^13^

There may be different reasons for the variability in mortality rates and associated factors, making it even more important to describe this in Ghanaian patients. The study aimed to describe the factors associated with diabetic ketoacidosis mortality among hospitalised patients from a tertiary hospital in Ghana.

## Methods

### Materials and Methods

We conducted a cross-sectional study of all 70 patients aged 18 years and older who were managed for DKA at the adult emergency room and adult medical wards of Korle-Bu Teaching Hospital from March to July 2019. Consecutive patients were enrolled over the period. The research team was alerted to any patient admitted and managed for DKA within the study period; all these patients were eligible for inclusion in this study. Consecutive patients were enrolled until the arbitrary number of 70 was reached, which was an estimate of the number of DKA patients expected to be seen within the period.

After discharge or death, folders of all patients who were managed for DKA were retrieved, and data were extracted into a data abstraction sheet. Baseline sociodemographic characteristics, as well as clinical and laboratory measurements at the time of presentation, were retrieved from the medical records.

Written consent was sought from all patients and next of kin for patients who died or were unconscious before their data was used for this study.

### Data Analysis

All data were collected in Microsoft Excel and imported into SPSS version 24 for analysis. Descriptive statistical analysis, including mean, standard deviation, and frequencies (percentages), was used to describe the study population. The Chi-square test or the Fisher exact test was used to determine associations between the categorical variables, where applicable. Univariate and multivariable binary logistic regression models were fitted to calculate crude odds ratios (OR) and adjusted odds ratios (aOR) with 95% confidence intervals (CI) to identify factors associated with mortality in the study population. The multivariable analyses were performed using the backwards selection method. Model 1 was adjusted using variables that were significant in the univariate logistic regression model. Model 2 was adjusted without including Glasgow Coma Score < 12. A p-value of < 0.05 was considered statistically significant.

The diagnosis of DKA was based on hyperglycaemia >11.0mmol/l, ketonuria (more than 2+) plus acidaemia of (pH<7.3) or bicarbonate (HCO3-) < 15.0mmol/L.^2^ However, in many instances, patients could not afford serum bicarbonate and pH testing; therefore, in addition to hyperglycaemia (> 11.0 mmol/L) and ketonuria (>2+), clinical signs of acidosis, such as Kussmaul breathing, aided in the diagnosis. We excluded patients with an osmolality greater than 320 mmol/L.

Ethical approval was obtained from the Ethical and Protocol Review Committee of the College of Health Sciences, protocol identification Number CHS-Et/M.5-4.1/2018/-2019.

## Results

The baseline demographic and clinical characteristics of the cases are presented in Table 1. A total of 70 patients were managed for DKA over the study period. The mean ages of patients who survived and those who died were 43.12 ± 15.81 and 49.09 ± 18.27, respectively (p = 0.266). Females accounted for 36 (51.4%) of the population, with most being under 39 years of age. The mortality rate was 15.7% (N=11), higher in females (81.8%) compared to their male counterparts (18.2%).

**Table 1:** Demographic and clinical characteristics of the study population

Parameters	Study population N=70 (%)	In-hospital survival N=59 (%)	In-hospital death N=11 (%)	P-value
**Age (years)**				
**Below 39**	32 (45.7)	27 (45.8)	5 (45.5)	0.255
**40-59**	24 (34.3)	22 (37.3)	2 (18.2)	
**60 and above**	14 (20.0)	10 (16.9)	4 (36.4)	
**Gender**				
**Male**	34 (48.6)	32 (54.2)	2 (18.2)	**0.046**
**Female**	36 (51.4)	27 (45.8)	9 (81.8)	
**Duration of diabetes**	7.19±6.0 4	6.2±4.5	10.7±9.5	0.230
**Undiagnosed diabetes**	32 (45.7)	29 (49.2)	3 (27.3)	0.208
**Type 2 diabetes^#^**	36 (51.4)	32 (65.3)	4 (50.0)	0.449
**Diastolic blood pressure, mmHg**	77.6±18.6	77.1±19.3	80.2±14.2	0.621
**Systolic blood pressure, mmHg**	124.9±24.5	123.4±23.0	132.7±31.6	0.253
**Systolic blood pressure < 90mmHg**	7 (10.0)	6 (10.2)	1 (9.1)	1.000
**Pulse rate, bpm**	107.7±19.8	105.8±20.0	118.1±15.6	0.057
**Pulse rate >100 or < 60 bpm**	43 (61.4)	33 (55.9)	10 (90.9)	**0.041**
**Temperature at presentation, °C**	37.0±1.0	37.0±1.0	36.9±1.2	0.783
**Temperature > 38 °C or < 36 °C**	17 (24.3)	14 (23.7)	3 (27.3)	1.000
**Blood glucose, mmol/l**	26.4±6.7	26.3±6.9	26.8±5.5	0.808
**Glasgow Coma Score**	14.4±1.5	14.5±1.2	13.4±2.3	0.125
**Glasgow Coma Score < 12**	8 (11.4)	4 (6.8)	4 (36.4)	**0.018**

# Diabetes status was available for 48 in-hospital survival patients and eight in-hospital patients who died

Of the 70 participants included in the study, 36 (51.4%) had type 2 diabetes. The numbers (proportions) of patients with type 1 diabetes, gestational diabetes, and Maturity Onset Diabetes of the Young (MODY) diagnosed based on clinical features before this index admission were 8 (11.4%), 4 (5.7%), and 1 (1.4%), respectively.

The proportion of Type 2 diabetes patients who survived (65.3%) was higher than those who died (50%), but the difference was not significant (p=0.449). Close to 90% of the patients who died had a pulse rate >100 or <60 bpm, significantly higher than those who survived (55.9%). The blood glucose levels on admission were comparable between the two study groups (p = 0.808). There was no significant difference in the mean diastolic and systolic blood pressures between the two groups. Although the mean pulse rate was higher in patients who died, the difference was not statistically significant (p = 0.057).

Two patients were managed in the Intensive Care Unit; only 6(8.6%) and 8(11.4%) of the patients had bicarbonate and blood pH done, respectively, and no patient was readmitted for recurrent DKA during this study period. Median length from admission to death was 2.5 (IQR, 9) days.

### Presenting complaints

The most common complaint at presentation was generalised weakness, accounting for about 30%. This was followed by impaired consciousness (25.7%), fever (14.3%), vomiting 12.9%, polyuria (11.4%) and other symptoms including cough, breathlessness, abdominal pain, chest pain and weight loss. The symptoms at presentation were not different between the survivors and non-survivors.

### Precipitating factors

**Figure 1:**
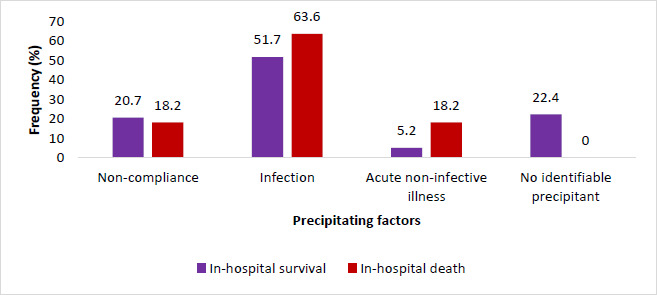
Precipitating factors of DKA

The precipitating factors for the two study groups have been presented in Figure 1. According to the chi-squared test, there was no significant association between precipitating factors and in-hospital outcomes (p = 0.1794). The most common precipitating factor was infection, accounting for 51.7% and 63.6% of DKA patients who survived and those who died, respectively. Similarly, the proportions of non-compliance as a precipitant of the DKA were comparable between the two groups. In about 20% of the in-hospital survivals, no precipitant was identified (Figure 1).

### Factors influencing mortality in the study population

The results of the univariate and multivariable logistic regression are presented in Tables 2 and 3. The positive association between age at diagnosis and mortality was not statistically significant. Infection was also not associated with in-hospital death in the DKA patients (OR = 1.633, 95% CI = 0.431-6.188, p = 0.470). In the univariate logistic regression analysis, female gender was associated with approximately a 5-fold increased risk of mortality.

**Table 2:** Univariate logistic regression analysis factors associated with mortality in diabetic ketoacidosis patients

Characteristics	Crude Odds of Dying	(95% CI)	p-value
**Age Z-score**	1.45	0.76 - 2.79	0.264
**Below 39 years**	1.00		
**40-59 years**	0.491	0.087-2.779	0.421
**60 and above**	2.160	0.481-9.697	0.315
**Female**	5.333	1.060-26.829	**0.042**
**Pulse rate >100 or < 60 bpm**	7.879	0.947-65.567	0.056
**Glasgow Coma Score < 12**	7.857	1.597-38.666	**0.011**
**Type 2 diabetes**	0.531	0.118-2.394	0.410
**Undiagnosed diabetes**	0.388	0.094-1.608	0.192
**Infection**	1.633	0.431-6.188	0.470

**Table 3:** Multivariable logistic regression models for in-hospital mortality in diabetic ketoacidosis patients

Characteristics	Model 1			Model 2		
	aOR	95% CI	p-value	aOR	95% CI	p-value
**Z-score age**	1.646	0.755-3.587	0.210	1.88	0.90-3.95	0.095
**Pulse rate >100 or < 60 bpm**	7.885	0.872-71.286	0.066	9.39	1.02-86.61	**0.048**
**Female sex**	4.271	0.672-27.159	0.124	6.31	1.11-35.83	**0.038**
**Glasgow Coma Score < 12**	3.390	0.483-23.809	0.220	-	-	-

Model 1 was adjusted by selecting all variables that were significant in the univariate logistic regression.

Model 2 was adjusted via backwards selection of variables without Glasgow Coma Score <12

Additionally, DKA patients with Glasgow coma score < 12 were about eight times more likely to die (OR=7.857; 95% CI, 1.597-38.666; p-value=0.011) (Table 2). In a multivariable logistic regression analysis, none of the factors was independently associated with mortality among DKA patients. In the adjusted logistic regression model via the backwards selection of the variables without Glasgow Coma Score < 12 (Table 4, model 2), female gender was associated with an over 6-fold increased risk of mortality, compared to their male counterparts (OR=6.31, 95% CI=1.11- 35.83, p=0.038). Pulse rate >100 or < 60 bpm was also independently associated with an over 9-fold increased risk of mortality among DKA patients (9.39 (1.02-86.61), p=0.048) (Table 3, model 2).

**Table 4:** Multivariable logistic regression models for in-hospital mortality in diabetic ketoacidosis patients

Characteristics	Model 1			Model 2		
	aOR	95% CI	p-value	aOR	95% CI	p-value
**Z-score age**	1.88	0.90-3.95	0.095	1.646	0.755-3.587	0.210
**Pulse rate >100 or < 60 bpm**	9.39	1.02-86.61	**0.048**	7.885	0.872-71.286	0.066
**Female sex**	6.31	1.11-35.83	**0.038**	4.271	0.672-27.159	0.124
**Glasgow Coma Score < 12**	-	-	-	3.390	0.483-23.809	0.220

Model 2 was adjusted via backwards selection of variables with Glasgow Coma Score <12

Model 1 was adjusted via backwards selection of variables without Glasgow Coma Score <12

## Discussion

This study provides important basic clinical information on mortality among patients admitted with DKA in a tertiary hospital in Ghana. We report a mortality rate of 15.7%. Female gender, Glasgow Coma Score < 12 and pulse rate >100 or <60 bpm was independently associated with mortality. The duration of diabetes, type of diabetes, blood pressure, temperature, blood glucose levels, and presence of infection were not associated with DKA mortality.

All patients were diagnosed based on hyperglycaemia, ketonuria, and clinical features, and only 6 (8.6%) and 8 (11.4%) of the patients had bicarbonate and blood pH tests, respectively. Additionally, only 2 out of 70 were managed in the ICU and none in HDU. The mortality rate of DKA patients in our study is higher than reported in studies in the USA and Europe.^7^-^9^ It is, however, similar to studies from Libya and Nigeria, with mortality rates of 11.7% and 18%, respectively.^11^,^12^ Our mortality rate is significantly lower than that reported in a study from Kenya, which found a mortality rate of 29.8%.^10^ but much higher than a study from Libya with a mortality of 0.6%.^13^ Possible factors contributing to the higher mortality in our study compared to their study include older age and fewer patients receiving ICU care. The patients in our study were relatively older (mean age of 44 years) than those from the Libyan study, with a mean age of 35.^13^ Older age has been associated with an increased risk of mortality in patients with DKA in previous studies.^14^, ^15^ Whilst only 2.85% of our patients were managed in the ICU, 91.6% of patients were managed in the ICU in the Libyan study. It is generally recommended that patients with DKA, particularly those with moderate to severe DKA, be managed in the ICU or High Dependency Unit (HDU) for optimal monitoring and management of fluid deficits, electrolyte imbalances, hyperglycemia, and acidosis.^16^ However, in most low to middle-income countries, ICU care, including bed availability, is minimal.^17^ and may even be unaffordable to many; this may increase the risk of complications and mortality in these patients^18^. Therefore, ICU or HDU must be made available to these patients in our setting.

It is important to note that, although pH and bicarbonate are integral in DKA diagnosis, only 6(8.6%) and 8(11.4%) of the patients had bicarbonate and blood pH done respectively and diagnosis was made based on 1) blood glucose > 11.0mmol/l; 2) significant ketonuria more than 2+ on standard urine sticks; and clinical features of acidosis in the absence of high osmolality. In our practice, blood gas analysers are readily available to ICU patients, but they come at a cost that is not always affordable to patients not admitted to the ICU. Another plausible reason for the differences in the mortality rates may be the precipitants of DKA. Some studies have reported that the higher mortality rate in their patients was due to infections^14^,^19^, which was not associated with our study.

The median duration from admission to death was 2.5 days. Other studies have suggested that DKA mortality is highest within the first 72 hours and it is during this period that patients ought to be managed in ICU.^20^ Only a small minority of our patients were managed in the ICU, which may have contributed to our high mortality.

The association between reduced Glasgow Coma Score (GCS) and DKA mortality has been demonstrated in numerous studies.^15^,^21^,^22^ In a retrospective study aimed at identifying predictors of 72-hour mortality in patients with diabetic ketoacidosis (DKA), reduced GCS was a strong predictor of 72-hour mortality.^21^ This was also reported in a Kenyan study, where altered consciousness in addition to impaired renal function on admission, was reported commonly in patients who died.^10^ Whilst low GCS may be indicative of a comorbid illness or precipitating illness such as a stroke, it may more importantly be indicative of severe DKA with coma. Thus, it was unsurprising to have seen a positive association with in-hospital mortality in this current study. The association was, however, attenuated in a fully adjusted regression model.

Gender disparity in DKA mortality has not been consistent. In this study, female gender was found to be associated with increased mortality. This was also suggested in a study from Kenya, where over 64% of patients who died were females.^10^ and also similar to a study in Thailand with a 3.5-fold higher mortality risk for female patients managed for DKA.^23^ These were consistent with a meta-analysis that showed that among critical patients admitted into the ICU, mortality was higher in females^24^ Although these patients were not necessarily patients with DKA, it suggests that in critical disease, females were likely to die. In contrast, in a large retrospective study of 25,627 DKA patients with a mortality rate of 3.3%, males were associated with in-hospital deaths.^15^

The positive association of female gender and mortality in DKA patients in this and other studies remain unclear, but existing evidence shows that females with diabetes present with much lower blood pH, indicative of severe DKA^25^ when diagnosed, acidaemia and its adverse systemic effects, including depression of neuronal, myocardial, and enzyme functions, could be a key driver of DKA mortality.^26^,^27^ Sex hormones oestrogen and progesterone have been shown to play a role in immunity and contribute to gender disparity in outcomes of many illnesses, but the exact correlation remains unclear.^28^

Pulse rate >100 or < 60 beats/minute was associated with mortality in our study. Tachycardia of > 100 may be a clinical feature of DKA, as dehydration from osmotic diuresis ensues, but may be related to hypovolaemic shock, a complication of DKA that may portend mortality. Severe bradycardia has been reported in DKA, and this may be due to electrolyte abnormalities present in DKA. Bradycardia may also be caused by severe acidosis; both of these may contribute to dysrhythmias that may increase mortality.^29^,^30^

This study is not without limitations; it is a cross-sectional study and is dependent on patient records; the sample size of 70 is also relatively smaller than other studies on DKA mortality^31^; therefore, the results may not be generalisable. However, although this small sample size may affect the effect size, this study provides useful baseline information on DKA mortality amongst Ghanaian patients.

Only 6(8.6%) and 8(11.4%) of the patients had bicarbonate and blood pH done, respectively. The inability of most patients to have pH and bicarbonate levels checked largely due to financial constraints is a limitation in this study, it may affect the diagnosis as we cannot be sure all these patients had DKA, it must be noted however that when pH and bicarbonate were not available, clinical features of acidosis were added to the other criteria to aid in diagnosis and hyperosmolality was ruled out. Although this is a limitation of this study, this may be the reality in many resource-challenged settings where blood gas analysers may not be readily available or are cost-prohibitive for patients, as in our case.

## Conclusion

In this study, the mortality rate of 15.7% was high compared to global mortality rates. Female gender, Glasgow Coma Score less than 12, and pulse rate >100 or <60 beats/minute were independently associated with mortality. The high mortality rates may have been due to gaps in diagnosis and the unavailability of ICU access.

To reduce mortality among patients admitted with DKA in the health care system of Ghana and similar settings, there is a need to improve diagnosis, investigations and management of DKA by using standardised protocols and guidelines. Additionally, there must be improved access to ICU and HDU services for DKA patients. Since this study was undertaken, the Korle Bu Teaching Hospital now has readily available ketone meters in the emergency room, which can be used objectively to assess the severity of DKA. Additionally, the National Guidelines for the Management of Diabetes Mellitus, the first comprehensive guidelines for diabetes management in Ghana, have been published^32^. We therefore recommend effective dissemination and use of these guidelines across the country and possibly a follow-up study to assess the impact of this guideline in the management of DKA.
